# Celecoxib decreases prostaglandin E_2 _concentrations in nipple aspirate fluid from high risk postmenopausal women and women with breast cancer

**DOI:** 10.1186/1471-2407-6-248

**Published:** 2006-10-18

**Authors:** Edward R Sauter, Wenyi Qin, Lisa Schlatter, John E Hewett, John T Flynn

**Affiliations:** 1 Surgery, University of Missouri, Columbia, MO 65212, USA; 2 Biostatistics, University of Missouri, Columbia, MO 65212, USA; 3 Physiology, Thomas Jefferson University, Philadelphia, PA 19107, USA

## Abstract

**Background:**

Celecoxib inhibits PGE2 production in cancerous tissue. We previously reported that PGE2 levels in nipple aspirate fluid (NAF) and plasma were not decreased in women at increased breast cancer risk who received celecoxib 200 mg twice daily (bid). The endpoints of the current study were to determine if a short course of celecoxib 400 mg bid would decrease PGE2 levels in women 1) at increased breast cancer risk, and 2) with established breast cancer.

**Methods:**

NAF and plasma samples were collected before, 2 weeks after taking celecoxib 400 mg bid, and two weeks after washout from 26 women who were at increased breast cancer risk. From 13 women with newly diagnosed breast cancer, NAF from the incident breast and plasma were collected before and on average 2 weeks after taking celecoxib. Additionally, in nine of the 13 women with breast cancer, NAF was collected from the contralateral breast.

**Results:**

No consistent change in NAF or plasma PGE2 levels was noted in high risk premenopausal women. NAF PGE2 levels decreased after celecoxib administration in postmenopausal high risk women (p = 0.02), and in both the NAF (p = 0.02) and plasma (p = 0.03) of women with breast cancer.

**Conclusion:**

Celecoxib 400 mg bid taken on average for 2 weeks significantly decreased NAF, but not plasma, PGE2 levels in postmenopausal high risk women, and decreased both NAF and plasma PGE2 levels in women with newly diagnosed breast cancer. PGE2 levels may predict celecoxib breast cancer prevention and treatment efficacy. Our observations are preliminary, and larger studies to confirm and extend these findings are warranted.

## Background

Women at increased breast cancer risk include those with a strong family history of breast cancer, those with a history of ductal carcinoma *in situ *(DCIS) or invasive breast cancer (IBC), and those with precancerous changes in their breasts. Eligibility for enrollment in the first National Surgical Adjuvant Breast and Bowel Project (NSABP) breast cancer prevention trial required that a participant have a > 1.66% projected 5 year probability of developing IBC [1]. The only accepted treatment for these women is tamoxifen, which has side effects of hot flashes, deep vein thrombosis, and uterine cancer. An effective alternative treatment is desirable.

Cyclooxygenase (COX)-1 and COX-2 may be present in breast tumors to catalyze the conversion of arachidonic acid to prostaglandins, prostacyclins, or thromboxanes. While COX-1 expression is constitutive, COX-2 is inducible [2] and is upregulated in a variety of tumors, including breast cancers [3]. Prostaglandin (PG)E_2 _is produced from arachidonic acid by either COX-1 or -2. PGE_2 _has tumor and cell growth promoting activity [4]. Women with breast cancer with PGE_2 _levels above 15 ng/g appear to have a significantly worse survival rate than those with levels ≤ 15 ng/g [5]. Malignant breast tumors produce more PGE_2 _than benign breast tumors or normal breast tissue [3]. A retrospective analysis of women with and without breast cancer seen at a single hospital for a one year period found that the women without breast cancer were more likely to have taken celecoxib than women who had developed breast cancer [6].

Nonsteroidal antiinflammatory drugs (NSAIDs), including aspirin, indomethacin and ibuprofen, inhibit both COX-1 and COX-2. Inhibition of COX-1 leads to a number of adverse effects, including gastrointestinal ulcers and renal toxicity [7]. Recent efforts have, therefore, focused on pharmacologic agents such as celecoxib, a clinically available medication which selectively inhibits COX-2. Preclinical studies suggest that celecoxib is effective both in preventing and in treating breast cancer in a dose dependant manner [8,9].

We are currently able to collect breast nipple aspirate fluid (NAF) from 95% of nonlactating adult females ages 18 to 80 with the use of a modified breast pump [10]. Using our NAF collection technique, we performed a preliminary study to determine if 200 mg bid of the COX-2 inhibitor celecoxib administered for two weeks to women at increased breast cancer risk would significantly decrease PGE_2 _levels in the breast, as measured both in NAF and in plasma, providing both organ specific and systemic information [11]. We observed that PGE_2 _is concentrated in NAF compared to matched plasma, but that celecoxib 200 mg bid administered for two weeks did not significantly decrease PGE_2 _concentrations in NAF or plasma.

Subjects with familial adenomatous polyposis (FAP) who received celecoxib for six months at 400 mg bid, but not those receiving 100 mg bid, demonstrated a significant reduction, compared to placebo, in mean polyp number [12]. Consequently, we sought to determine if a short two week course of celecoxib at a dose of 400 mg bid would alter NAF and/or plasma PGE_2 _levels in women at high risk for, or with newly diagnosed breast cancer. We observed that celecoxib 400 mg bid lowered PGE_2 _levels in the NAF and plasma of women with breast cancer, and in the NAF of high risk postmenopausal women.

## Methods

### Subject recruitment

Women were provided an Institutional Review Board approved protocol and required to give written informed consent in order to enroll in the study. Subjects evaluated had to be ≥ 18 years old and be at increased breast cancer risk, based on the subject having either a Gail model risk of developing IBC in a 5 year period of > 1.66%, or previously treated DCIS or IBC (now finished with treatment and free of disease).

Pregnant and lactating women were not eligible. Women could not have been currently on NSAIDs, aspirin, a COX-2 inhibitor, warfarin, or have taken such a medication within two weeks of enrollment. Subjects could not have a significant history of peptic ulcer disease, upper gastrointestinal bleeding, asthma, or be allergic to sulfonamides or NSAIDs. A complete blood count, serum electrolytes and liver panel had to be within normal limits. Subjects were recruited from the Breast Evaluation Clinics at the University of Missouri-Columbia.

### Intervention

Celecoxib was taken daily for 14 days by women at increased risk, and for 10 to 24 (median 14) days by women with recently diagnosed breast cancer. Compliance was assessed through the count of returned pills. All subjects were required to have taken at least 80% of the prescribed medication. While the goal was to have women at increased risk and women with cancer take celecoxib for the same time period, variability in the operative date required some flexibility in the latter group.

### Specimen collection

For all women, both those with cancer and those at increased risk, NAF was analyzed from only one breast. If one breast contained cancer and the other did not, only NAF from the breast with cancer was analyzed. For serum collected from women with cancer, PGE_2 _values were assigned to the cancer group. For all subjects, NAF was collected from the same breast for each of the three visits. A detailed account of the NAF collection technique has been reported [10,13].

Baseline NAF and blood collection were performed prior to the ingestion of celecoxib. For women at increased risk, washout NAF and blood samples were collected 14 days after stopping celecoxib. Thus, subjects at increased risk were asked to provide three NAF and three plasma samples (baseline, after celecoxib, and after washout), whereas subjects with recently diagnosed cancer provided two NAF and two plasma samples. Each subject granted permission for us to contact them yearly to determine if they had developed new or recurrent breast cancer.

NAF samples were collected into capillary tubes and stored at -80°C until analysis. Eight mL of blood were also collected from the subject in a tube containing heparin, the blood spun for 10 min at 1600 rpm, and the plasma fraction decanted and stored at -80°C until analysis. All women had NAF and plasma collected within 12 hrs of their last dose of celecoxib. The half life of the medication is 11.5 hrs.

### PGE_2 _analysis

The biomarker chosen for analysis was PGE_2_, due to its established link to cancer growth. NAF and plasma samples were analyzed by immunoassay for their PGE_2 _content. NAF and plasma samples were analyzed as per the manufacturer's instructions (R&D Systems, Minneapolis, MN). The kit uses a monoclonal antibody to PGE_2 _to competitively bind the PGE_2 _in the standard or sample. Briefly, samples were diluted in 100 μL assay buffer supplied by the manufacturer, pipetted into appropriate wells, incubated for 18–24 hrs at 4°C, washed, substrate solution added, followed by 1 hr incubation, and absorbance measured at 405 nm.

For NAF and plasma analyses, a standard curve was prepared using serial dilutions of PGE_2_. A linear regression equation was created from standards of known PGE_2 _concentration, and PGE_2 _concentrations of unknown samples fit to the standard curve regression equation, corrected for aliquot volume and expressed as nanograms of PGE_2_/mL of original sample. The goodness of fit of the standard curve, R^2^, for NAF samples was 0.999. The goodness of fit was similar for the plasma samples.

### Statistical analysis

Median values of continuous variables were computed for the various groups of subjects. Due to the potential non-normality of the data, ranking procedures were used for all analyses with continuous variables. The Wilcoxon Rank Sum Test was used to compare independent groups. Examples of these comparisons include comparing pre- and postmenopausal women, comparing the cancer to the high risk group, etc. The Wilcoxon Signed Ranks Test was used to make within group comparisons such as comparing pretreatment to posttreatment, pretreatment to washout, etc.

## Results

### Subjects

Subjects were enrolled from May 2003 to December 2004. Complete recruitment data were available for all subjects screened in 2004, during which the majority of the subjects analyzed in the study were recruited. In 2004, 158 women were screened, of which 27 were eligible, enrolled and were evaluable. Reasons for ineligibility included use of nonallowed medication, medical history, and blood screen abnormalities. All eligible subjects were evaluable, which required successful collection of NAF or plasma and detection of PGE_2 _before and after treatment in the samples.

NAF and plasma samples were collected before, 2 weeks after taking celecoxib 400 mg bid, and two weeks after washout from 26 women who were at high risk for developing breast cancer. NAF was successfully collected from the same breast at for 95% (113/120) of subject visits. From 13 women with newly diagnosed cancer, NAF from the incident breast and plasma were collected before and on average 2 weeks after taking celecoxib 400 mg bid. Additionally, in nine of the 13 women with breast cancer, NAF was collected from the contralateral breast. Thus, we collected 26 NAF and 26 plasma samples from the 26 women at increased risk of breast cancer, plus 9 NAF samples from breasts contralateral to a breast with cancer, for a total of 35 NAF samples from breasts at increased cancer risk. We collected NAF from 12/13 subjects from the breast containing cancer, as well as plasma in the 13 subjects with cancer (Table 1). In total, 47 NAF and 39 blood samples were collected at baseline from 39 subjects.

Fewer than half of subjects were premenopausal in both the high risk and cancer groups. Median age was lower in high risk subjects than in subjects with breast cancer. All but one subject recruited was Caucasian. The median number of celecoxib pills taken was over 95% by subjects in both the high risk and cancer groups, and all subjects in both groups took over 80% of the pills that they were given.

### PGE_2 _concentration decreases in both NAF and plasma of women with breast cancer after celecoxib treatment

**Figure 1:**
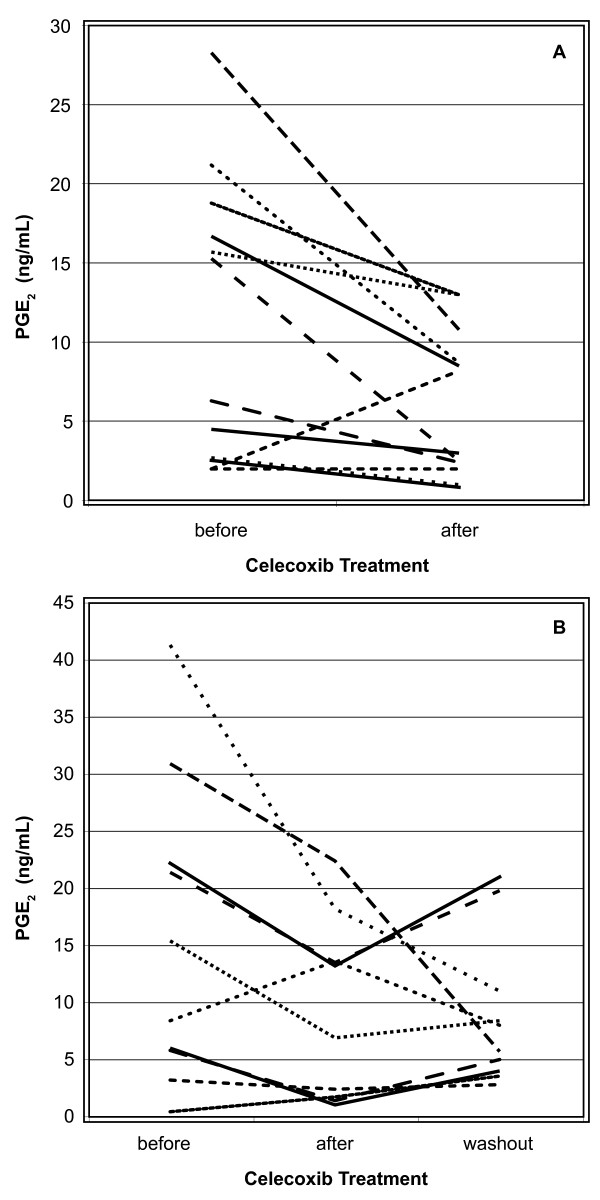
PGE_2 _values in NAF from A) women with breast cancer, and B) high risk postmenopausal women. Only subjects with before and after treatment values (cancer subjects) or with before, after and washout values (high risk subjects) are shown.

We evaluated all NAF samples for PGE_2 _(Table 2). PGE_2 _levels were not significantly different when comparing subjects at increased risk to women with cancer. When evaluating the effect of celecoxib on PGE_2_, levels did not significantly change in high risk women, but decreased significantly among women with newly diagnosed breast cancer (p = 0.02). Changes in individual subjects after treatment are illustrated in Figure 1A.

**Figure 2:**
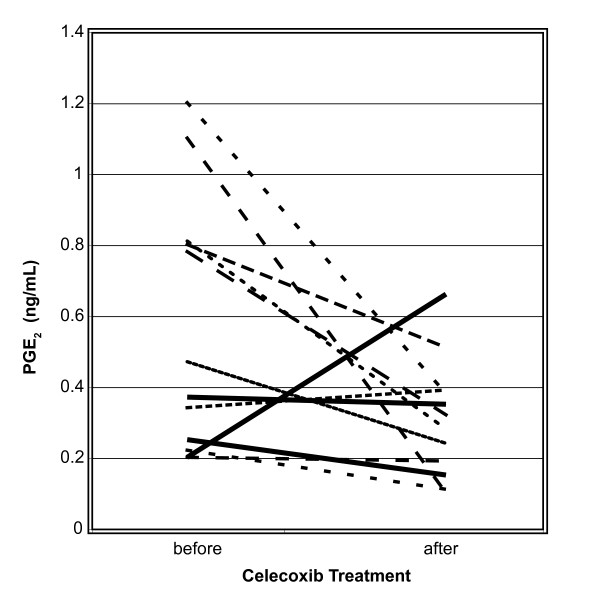
PGE_2 _values in plasma from women with breast cancer. Only subjects with before and after treatment values are shown.

PGE_2 _concentrations in plasma were determined in all samples (Table 3). Similar to findings in NAF, PGE_2 _levels were not significantly different when comparing samples from subjects at increased risk to women with cancer. When evaluating the changes in PGE_2 _before vs. after treatment, levels did not significantly change in high risk women, but decreased significantly in samples from women with newly diagnosed breast cancer (p = 0.03). Changes in individual subjects after treatment are illustrated in Figure 2.

### Celecoxib decreases PGE_2 _levels in the NAF and plasma of postmenopausal women

We next determined if menopausal status influenced the effect of celecoxib on PGE_2 _levels (Tables 4 and 5). Celecoxib significantly decreased PGE_2 _levels in the NAF (p = 0.02) but not the plasma of postmenopausal high risk women. Changes in individual subjects after treatment and after washout are illustrated in Figure 1B. Celecoxib did not significantly alter PGE_2 _levels in premenopausal women.

### PGE_2 _levels are higher in pre- than in postmenopausal high risk women

Both after treatment (p = 0.04) and after washout (p = 0.003), NAF PGE_2 _levels were significantly higher in pre- than in postmenopausal high risk women (Table 4). PGE_2 _levels in NAF were not different in women with newly diagnosed breast cancer. After washout, plasma PGE_2 _levels were higher (p = 0.04) in pre- than in postmenpausal women (Table 5). No differences in plasma PGE_2 _levels were found, either before or after treatment, in women with newly diagnosed breast cancer. Moreover, NAF and plasma PGE2 levels were not correlated in either the cancer or high risk groups, whether or not they were subdivided by menopausal status.

### PGE_2 _is concentrated in NAF compared to matched plasma

When both NAF and plasma were available from the same subject, relative concentrations of PGE_2 _were compared before and after treatment with celecoxib. In the high risk (cancer) groups, median concentrations of PGE_2 _in NAF were 27.9 (16.4), 63.7 (13.9), and 48.1 (not applicable) times higher than in corresponding plasma before celecoxib, after celecoxib, and after washout. The differences in the ratios of NAF to serum PGE_2 _levels before vs. after treatment, before vs. after washout, and after treatment vs. after washout were significant in neither the high risk nor the cancer group.

### Toxicity

In the high risk group, there were 11 subjects who experienced side effects from celecoxib, four of whom dropped out. In the other 7 subjects, the side effects resolved spontaneously. In the four who dropped out, the side effects (edema in two, diarrhea in one, and heart palpitations in one) all resolved shortly after stopping celecoxib. Of the 13 subjects with recently diagnosed cancer who received celecoxib, one had insomnia(this subject is also included in the at risk group, since NAF was collected from the non-involved breast) which resolved spontaneously. There were no dropouts in the cancer group.

## Discussion

We carried out experiments to investigate whether the COX-2 inhibitor celecoxib at a dose of 400 mg bid could significantly affect endogenous levels of PGE_2 _in NAF or plasma of women at risk for breast cancer or in women with known cancer. This study builds on our initial study, which found that celecoxib 200 mg bid did not alter PGE_2 _levels in NAF or plasma under normal, nonstressed conditions.

In the current study, we observed two important findings. First, PGE_2 _levels significantly decreased in the NAF, but not in the plasma, of postmenopausal women at increased risk of breast cancer. Second, PGE_2 _levels significantly decreased in both the NAF and plasma of celecoxib-treated women with newly diagnosed breast cancer.

It is unclear why PGE_2 _levels significantly decreased in post- but not premenopausal high risk women. We did not routinely record whether premenopausal subjects started celecoxib in the first or second half of their menstrual cycle. We have this information for 10 women in the high risk group and one woman for the cancer group. We did not find a significant difference in PGE_2 _response to celecoxib in either the high risk or cancer group based on when the subject started medication in the first or second half of her cycle, although sample size limits the reliability of these findings.

In order to assess if PGE_2 _was concentrated in NAF, we determined its relative concentration in NAF vs. plasma in both high risk women and in women with newly diagnosed breast cancer. In both groups, PGE_2 _was concentrated in NAF relative to plasma. The NAF/plasma relative concentration was numerically, although not significantly, lower in women with cancer than in those at increased risk.

The relative concentration of PGE_2 _in NAF vs. plasma differed in the current study compared to our earlier study [11]. There are a number of possible reasons for this. First, relative concentrations were lower in the cancer group, which was not present in the earlier study. Second, we used different kits in the two studies. The kit used in the current study was chosen because it has been shown capable of measuring PGE_2 _in human bodily fluids by other investigators [14]. Third, we used a higher dose of celecoxib (400 mg bid vs. 200 mg bid) in the current study.

COX-2 expression has been evaluated in preclinical models and in clinical breast specimens. In a dimethylbenzanthracene (DMBA) rat model of breast cancer, celecoxib was found to dramatically reduce the incidence, multiplicity, and volume of breast tumors relative to control [15]. While COX-2 expression appears to be a good marker of breast cancer in tissue, the lack of reliable commercial immunoassays for bodily fluids at the time that the study was conducted limited our ability to measure this marker in NAF and plasma.

## Conclusion

PGE_2 _is measurable and is concentrated in NAF compared to plasma. Plasma concentrations of PGE_2 _in women with a predisposition to breast cancer are within the normally accepted basal range (less than 1 nanogram/mL). Celecoxib 400 mg bid significantly decreased PGE_2 _levels in women with newly diagnosed breast cancer in both NAF and plasma, and in the NAF of high risk postmenopausal women, consistent with clinical studies evaluating its effects on colon and duodenal polyps. We acknowledge that additional studies, with a placebo control, are required to determine whether monitoring NAF eicosanoid concentrations will be of value in describing the development and progression of breast cancer, or in monitoring the effect of candidate chemopreventive agents such as celecoxib.

## Abbreviations

ADH: atypical ductal hyperplasia; DCIS: ductal carcinoma *in situ*; DMBA: dimethylbenzanthracene; FAP: Familial Adenomatous Polyposis; IBC: invasive breast cancer; NAF-nipple aspirate fluid; NSABP: National Surgical Adjuvant Breast and Bowel Project; NSAIDs: nonsteroidal anti-inflammatory drugs; PG: prostaglandin

## Competing interests

The author(s) declare that they have no competing interests.

## Authors' contributions

ERS designed the study, enrolled subjects, and performed the majority of manuscript preparation. WQ conducted all PGE2 analyses. JTF assisted with manuscript preparation and critique. LS enrolled subjects and entered data. JEH performed the statistical analyses.

## Pre-publication history

The pre-publication history for this paper can be accessed here:



## Figures and Tables

**Table 1:** 

	**Demographics**	
	**High Risk**	**Cancer**
	**NAF(plasma)**	**NAF(plasma)**
Samples	35(26)^1^	12(13)^2^
**Age (years)**		
Median	53(50.5)	59(58)
Range	30–81(30–81)	35–77(35–77)
**Premenopausal**	14(11)	3(3)
**Race**		
White	34(25)	12(13)
Black	0	0
Asian	0	0
American Indian	1(1)	0
**Primary risk factors for enrollment**		
Family history	17(17)	0
Atypia	6(6)	0
History of breast cancer	3(3)	0
New contralateral breast cancer	9(0)	0
New ipsilateral breast cancer	0	12(13)
**Concomitant medications**		
Vitamins	14(12)	3(3)
Alternative therapies	7(4)	1(2)

1: NAF was collected from both breasts of nine women who had cancer diagnosed on one side. NAF from the incident breast was placed in the cancer group and from the contralateral breast in the high risk group. Plasma from these women are included only in the cancer group.

2: We were unable to collect NAF from one cancer subject at baseline.

**Table 2:** Comparison of Median PGE_2 _Concentrations in ng/mL in NAF Based on Disease Status^1^

	***Before (N)***	***After (N)***	***Washout (N)***	***P value***		
				
				**Before v.** **After (N)**	**After v.** **Washout (N)**	**Before v.** **Washout (N)**
**Population**^2^						
**High risk**	11.43 (26)	13.22 (22)	14.30 (22)	0.84 (22)	0.91 (21)	0.67 (22)
**Cancer**	10.70 (12)	8.11 (11)	NA	**0.02 (11)**		
***P value***	0.37	0.11				

1: N: sample size; NAF: nipple aspirate fluid; NA: not applicable.

2: Although NAF was collected from both breasts of women with cancer, only NAF from the breast with cancer is included in this Table. In the high risk group, after taking celecoxib NAF collection was unsuccessful in two subjects, and in one the tube broke. In the cancer group, one subject refused NAF collection, and in a second subject the after treatment aspiration was not attempted due to the presence of a needle localization wire.

**Table 3:** Comparison of Median PGE_2 _Concentrations in ng/mL in Plasma Based on Celecoxib Dose and Disease Status^1^

	***Before (N)***	***After (N)***	***Washout (N)***	***P value***		
				
				**Before v.** **After (N)**	**After v.** **Washout (N)**	**Before v.** **Washout (N)**
**Population**^2^						
**High risk**	0.29 (26)	0.21 (26)	0.25 (23)	0.72 (26)	0.50 (23)	0.70 (23)
**Cancer**	0.37 (13)	0.30 (12)	NA	**0.03 **(12)		
***P value***	0.16	0.99				

1: N: sample size; NA: not applicable.

2: Reasons why blood was not collected: In the high risk group at washout, three subjects refused. In the cancer group, one subject refused blood collection after treatment.

**Table 4:** Effect of Menopausal Status on Median PGE_2 _Concentrations in ng/mL in NAF^1^

	***Before (N)***	***After (N)***	***Washout (N)***	***P value***		
				
				**Before v.** **After (N)**	**After v.** **Washout (N)**	**Before v.** **Washout (N)**
**Menopausal Population**						
***High risk***						
Pre-	15.72 (11)	36.06 (11)	27.64 (11)	0.17 (11)	0.76 (11)	**0.04 **(11)
Post-	5.88 (15)^2^	6.81 (11)	5.57 (11)	**0.03 **(11)	0.77 (10)	**0.03 **(11)
***P value***	0.18	**0.04**	**0.003**			
***Cancer***						
Pre-	15.2 (3)	2.40 (3)	NA	0.25 (3)		
Post-	10.9 (8)	8.27 (8)	NA	0.11 (8)		
***P value***	1.00	0.61				

1: Abbreviations: NAF: nipple aspirate fluid; N: sample size; NA: not applicable.

2: Before treatment median PGE_2 _for the 11 subjects with an after treatment comparison value was 8.33, which was significantly higher than the after treatment group.

**Table 5:** Effect of Menopausal Status on Median PGE_2 _Concentrations in ng/mL in Plasma^1^

	***Before (N)***	***After (N)***	***Washout (N)***	***P value***		
				
				**Before v.** **After (N)**	**After v.** **Washout (N)**	**Before v.** **Washout (N)**
**Menopausal Population**						
***High risk***						
Pre-	0.37 (11)	0.37 (11)	0.45 (10)	0.77 (11)	0.61 (10)	0.77 (10)
Post-	0.28 (15)	0.19 (15)	0.18 (13)	0.64 (15)	0.59 (13)	0.45 (13)
***P value***	0.53	0.47	**0.04**			
***Cancer***						
Pre-	0.25 (3)	0.35 (3)	NA	0.99 (3)		
Post-	0.78 (9)	0.28 (9)	NA	**0.01 **(9)		
***P value***	0.27	0.58				

1: Abbreviations: N: sample size; NA: not applicable
